# Normal reference values for Tc-99m pertechnetate thyroid uptake in northern Iranian population: A single-center experience 

**DOI:** 10.22088/cjim.15.3.459

**Published:** 2024-08-01

**Authors:** Amir Gholami, Nima Armaghan, Hoda Shirafkan, Mehrangiz Amiri, Seyyed Hossein Mousavie Anijdan

**Affiliations:** 1 1.Department of Radiology, School of Medicine, Babol University of Medical Sciences, Babol, Iran; 2 Babol University of Medical Sciences, Babol, Iran; 3 Social Determinants of Health Research Center, Health Research Institute, Babol University of Medical Sciences, Babol, Iran; 4 Department of Radiation Technology, Allied Medicine Faculty, Babol University of Medical Sciences, Babol, Iran

**Keywords:** Thyroid uptake, Tc-99m pertechnetate, Hyperthyroidism, Radioiodine

## Abstract

**Background:**

The normal reference values for the thyroid uptake of radioactive iodine and Tc-99m pertechnetate in euthyroid patients vary by geographical location as well as the amount of iodine intake in the diet. The present study examines the normal reference values for thyroid uptake of Tc-99m pertechnetate in the North of Iran.

**Methods:**

The participants of this study were 64 patients (all over 20 years of age) who had referred to the Nuclear Medicine Center of the Shahid Beheshti Hospital for thyroid scan over the period between March 2018 and May 2020. It is worth mentioning that relying on laboratory test results, only patients with normal thyroid function were included in this cross-sectional study.

**Results:**

The median, the 5^th^ and 95^th^ percentiles and thyroid uptake range of 99mTc-pertechnetate in euthyroid patients were 0.9, 0.6 to 1.8% and 0.54 - 1.80%, respectively.

**Conclusion:**

The percentage of uptake in the thyroid gland in each geographical area varies based on race and diet content, so it is necessary to determine the percentage of uptake in each specific region and even check it periodically.

The thyroid uptake and scintigraphy play an essential role in the diagnosis of thyroid diseases and abnormalities in thyroid function. Iodine-131, discovered in the late 1970s, was the first radioisotope used to assess thyroid uptake. Since then, the measurement of thyroid uptake and thyroid gland imaging has been used as an effective tool to diagnose thyroid disorders. Various etiologies of hyperthyroidism are differentiated via the measurement of Iodine-131 uptake (RAIU). Also, thyroid scintigraphy is valuable in assessing hyperthyroidism, thyroid nodules, differentiated thyroid carcinoma, and detecting abnormal thyroid tissue (1, 2). 

By measuring RAIU, we can differentiate hyperthyroidism from other causes of thyrotoxicosis. Patients with subacute thyroiditis often exhibit a diffuse pattern of decreased uptake, while in patients with Graves' disease or toxic nodular goiter, increased uptake is often seen (3-5). Since iodine-131 has serious drawbacks related with high doses of radiation, its use is confined to staging and the follow-up of patients with thyroid cancer. Iodine-123 is a suitable alternative to iodine-131, which is due to its shorter half-life and desirable energy for imaging; unfortunately, it is not generally available, especially in Iran, due to its high production cost. 

Nowadays, Tc-99m pertechnetate has become the most common radiotracer for thyroid scanning, which is due to its low cost, easy access, short half-life (6 hours), short time from injection to imaging, less radiation to the patient (10000 time less than that of I-131), and suitable preferred gamma energy (140 keV) for imaging.

Thyroid gland absorbs Tc-99m pertechnetate ions and iodide ions in the same pathway. Even though the thyroid does not organize Tc-99m pertechnetate, estimation of the uptake and imaging provide all the information necessary for accurate diagnosis (6-8). 

It is generally stated that the uptake of Tc-99m pertechnetate is a beneficial way to diagnose and differentiate the causes of thyrotoxicosis, especially Graves' disease, from destructive thyroiditis, but there is no absolute criterion for making such a diagnosis (9). 

The normal reference values for the thyroid uptake of radioactive iodine and Tc-99m pertechnetate in euthyroid individuals vary by geographical location and the amount of iodine intake in their diet. It may also vary in different decades. Over time, natural fluctuations in reference values may occur, indicating the significance of periodical evaluation of normal reference values for thyroid uptake (6, 10-14). It is generally assumed that the reference values specific to a specific geographic area for thyroid uptake of Tc-99m pertechnetate are infrequent. Hence, there is an urgent need to define the reference values for each geographical location. It is commonly believed that each clinical laboratory should establish its own criteria for defining the normal range, as the uptake of Tc-99m pertechnetate depends on the technique used (15). There is no report about uptake of Tc-99m pertechnetate in the geographical location where we live, North of Iran; we determined the normal reference values for thyroid uptake of Tc-99m pertechnetate in euthyroid individuals in this study. 

## Methods

Sixty-four patients (31 males and 33 females) who had referred to the Nuclear Medicine Center of the Shahid Beheshti Hospital over the period between March 2018 and May 2020 participated in this study. The participants were over 20 years of age and had normal thyroid function confirmed by laboratory test results. In addition, the study was conducted under the approval of the Babol University of Medical Sciences Research Ethics Board, IR.MUBABOL.REC.1399.026. 

There was no history of thyroid disorders in all patients based on the history taken. Moreover, patients with a history of heart, kidney, and liver diseases were excluded from the study.

 Patients with recent iodine-contrast radiographs or those taking drugs affecting thyroid function were also excluded. All female participants were not pregnant, nor were they breastfeeding at the time of the study. It should be stated that none of the participants were taking tobacco, dietary supplements, or medications containing vitamins and minerals. Also, no goiter or thyroid nodule was touched on the physical examination of the thyroid gland in patients, which was subsequently confirmed after a thyroid scan.

The laboratory evaluation of thyroid function was performed by serum measurements of thyroid stimulating hormone (TSH), triiodothyronine (T3) and thyroxin (T4). All patients were injected by Tc-99m pertechnetate at a dose of 5 mCi. The percentage uptake of Tc-99m pertechnetate was determined through the thyroid gland in 20 minutes using nuclear imaging techniques.

 For this purpose, a gamma camera (Siemens, Orbiter ZLC 75) equipped with a low-energy parallel hole collimator was used. Images were acquired in a matrix with 128 × 128 pixel and a zoom of 1.5. Images were taken from the syringes pre and post injection of Tc-99m pertechnetate to determine the precise quantity of the prescribed dose to the patients. Images were taken from the syringe as well as the front of the neck for 60 seconds to show the thyroid gland. Finally, the number of syringes counted before and after the injection was obtained from the images. A controlled image of the injection site is normally taken for the accuracy of the intravenous injection because extravasations are generally unacceptable for calculating the percentage of thyroid uptake.

**Figure 1:**
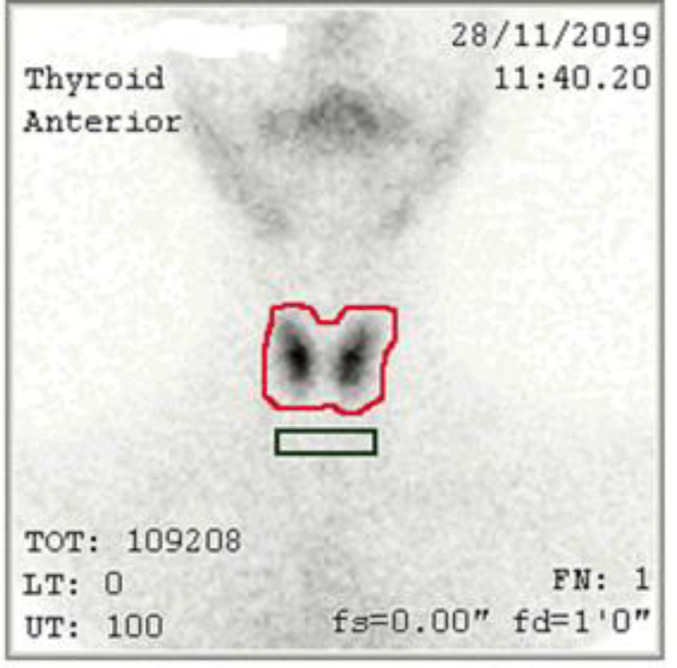
Determining the location of the thyroid gland by drawing a red line around it and determining the background tissue as a green rectangle

The total number of thyroid counts was calculated via determining the thyroid boundaries on the images through drawing an irregular region of interest and also by drawing a rectangular area with a length almost equal to the thyroid gland to determine the background count (figure 1). Thyroid uptake of Tc-99m pertechnetate is calculated for each participant by means of the following formula:

$\mathrm{\% uptake}=\frac{\mathrm{Thyroid count}–\mathrm{Background count}}{\mathrm{pre injection syring count}-\mathrm{post injection syring count}}$

We analyzed the data using SPSS software Version 21. Descriptive analysis was presented using mean, median and standard deviation as well as frequencies and percentages. T-test was used to compare the percentage of uptake, TSH, T3 and T4 in the population. Pearson correlation analysis was applied to survey the correlation between uptake, age and sex of patients. The data normality was checked by the Shapiro-Wilk test. A significance level was considered less than 0.05 (p<0.05).

## Results

A total number of 64 (31 males and 33 females) patients with the mean age of 39.48 ± 14.97 participated in this study. All patients had normal thyroid function tests. The mean results of thyroid hormone levels; including TSH, T3 and T4 are given in table 1, which were all in the normal range (table 1). The mean ± SD percentage of thyroid Tc-99m pertechnetate uptake was 1.09 ± 0.33 in men. It was 0.94 ± 0.33 in women and also 1.01 ± 0.33 in all patients (table 2). 

**Figure 2:**
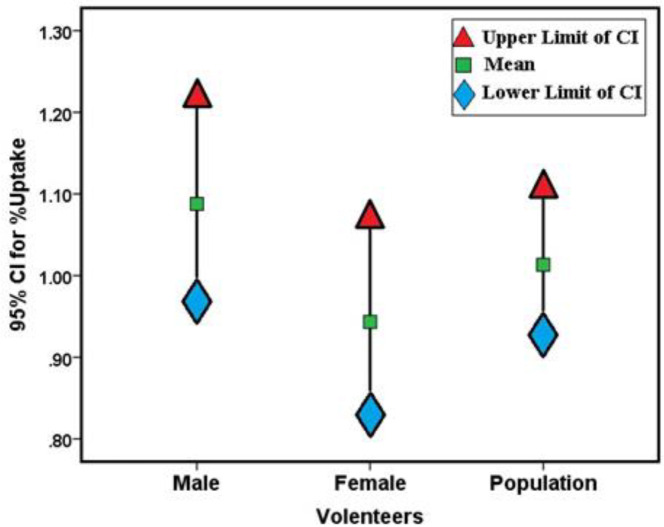
Gender relationship of Tc-99m pertechnetate uptake in euthyroid participants

**Figure 3:**
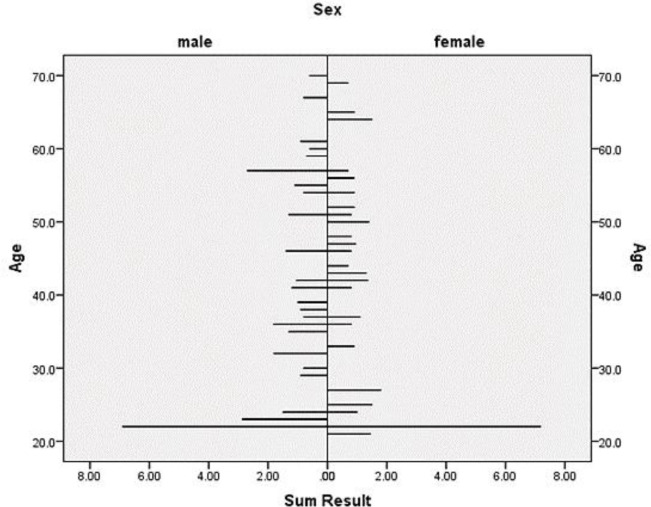
Comparison of age of Tc-99m pertechnetate uptake in euthyroid participants

**Figure 4:**
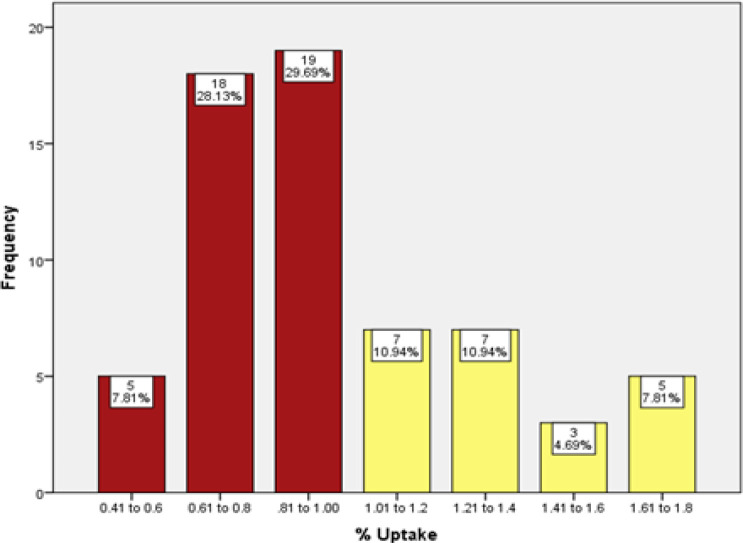
Frequency histogram of percentage uptake of Tc-99m pertechnetate in euthyroid participants

In figure 2, the percentage of uptake in each gender group was compared with the uptake of all patients participating in the study. The t-test showed that gender did not appear to play an important role in thyroid uptake Tc-99m pertechnetate in euthyroid individuals (P= 0.083), but the normal values were slightly lower in women (0.94 ± 0.33) compared with those of men (1.09 ± 0.33). Figure 3 shows the percentage of uptake of Tc-99m pertechnetate in male and female groups participating in this study by age. In both groups, the amount of uptake decreased with age. To conclude whether the uptake percentage of Tc-99m pertechnetate is normally distributed among the population of our geographical area, the percentages of uptake were divided into groups with uptake intervals of 0.2%, and a histogram of the average uptake in different groups was accordingly plotted (figure 4).

**Table 1:** Demographic characteristics and information of thyroid function hormones in euthyroid participants

**T4** **Mean ± SD**	**T3** **Mean ± SD**	**TSH** **Mean ± SD**	**Min - Max (years)**	**Mean Age (years)**	**Total**	**Group**
8.32 ± 0.80	1.60 ± 0.15	2.50 ± 1.14	21 - 69	39.2 ± 14.8	33	**Female**
8.27 ± 0.46	1.57 ± 0.16	2.78 ± 0.81	22 - 70	39.8 ± 15.4	31	**Male**
8.30 ± 0.08	1.58 ± 0.02	2.63 ± 0.12	21 - 70	39.48 ± 14.97	64	**Population**
4.7-12.5 ug/dL	0.6-2.1 ng/mL	0.3-5.2 mIU/mL	-	-	-	**Normal Laboratory Range**

**Table 2:** Range, mean and median uptake of Tc-99m pertechnetate in euthyroid participants

**% Uptake 95% CI**	**% Uptake Mean ± SD**	**% Uptake Min - Max**	**Median**	**Total**	**Group**
0.83 - 1.06	0.94 ± 0.33	0.54 - 1.80	0.89	33	**Female**
0.97 - 1.21	1.09 ± 0.33	0.6 - 1.80	1.00	31	**Male**
0.93 - 1.09	1.01 ± 0.33	0.54 - 1.80	0.9	64	**Population**

**Figure 5:**
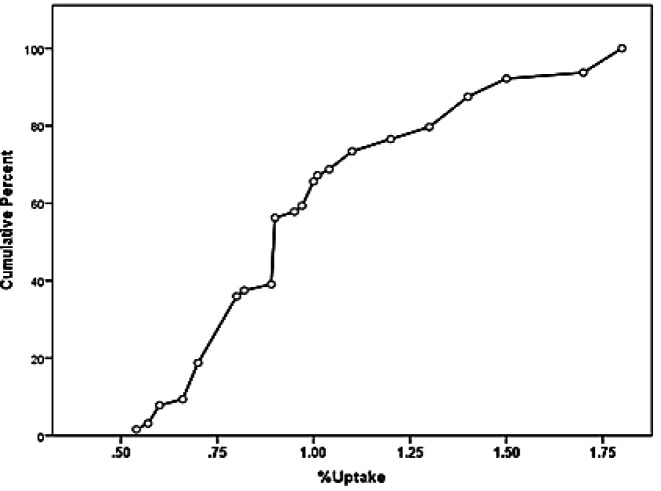
Cumulative frequency diagram of Tc-99m pertechnetate uptake in participants in the range of 5^th^ and 95^th^ percentiles

This histogram clearly showed that the percentage of uptake in our study population was not normally distributed (p<0.001). Since the data did not follow the normal distribution, the values of 95% CI and mean ± SD were not suitable for determining the normal range of uptake. To calculate the reference range for the percentage uptake of Tc-99m pertechnetate, the data from figure 4 is shown as a cumulative frequency curve in figure 5. The 5^th^ and 95^th^ percentiles were used to determine the normal uptake reference range. The 5^th^ and 95^th^ percentiles were calculated from 0.6 to 1.8% in this study. 

## Discussion

Nowadays, Tc-99m pertechnetate is broadly used as the radiopharmaceutical choice in thyroid scintigraphy. It is also believed that the absolute uptake of Tc-99m pertechnetate through the thyroid gland is low. Owing to the fact that the amount of uptake in the thyroid depends on the geographical area as well as the iodine content of the diet, different percentages of uptake have been expressed in different studies (table 3). Consequently, determining the range of percentage thyroidal uptake of Tc-99m pertechnetate in each specific geographical area is a great help in interpreting thyroid scintigraphy, especially in determining the cause of thyrotoxicosis (6, 12-14).

**Table 3:** Normal values for uptake of Tc-99m pertechnetate in the literature

**Range** ** (%)**	**Year**	**Country**		
0.5–4.0	1968	USA	**Atkins and Richards**	**1**
0.4–3.0	1968	UK	**De Garreta et al.**	**2**
0.24–3.4	1972	USA	**Hurley et al. **	**3**
0.7–2.9	1972	UK	**Van’t Hoff et al.**	**4**
0.4–1.7	2002	Brazil	**Ramos et al.**	**5**
0.15–1.69	2013	Namibia	**Hamunyela et al.**	**6**
0.2–2	2018	UK	**Macauley et al.**	**7**

In our study, the reference uptake values were recognized to range from 0.6 to 1.8%. The lower uptake threshold of our study is similar to those of most studies in this field. The upper uptake threshold in this study seems to have decreased significantly compared to those of older studies (1960s-1980s) but it has been very similar to the uptake thresholds of more recent studies. Brazil, for instance, ranged from 0.4 to 1.7 in 2002, Namibia ranged from 0.15 to 1.69 in 2013, and England ranged from 0.2 to 2 in 2018 (10-12). This declining trend in thyroid uptake over the past half-century could be due to the planned increase in dietary iodine content and the increased use of seafood. Due to the decline of high uptake threshold in our study and with regard to other studies in other areas of the world, it seems that the prevalence of iodine deficiency in our region has decreased. One important reason could be the daily intake of iodized salt. The results also depicted that there was a negative correlation between the percentage uptake and age in our study (figure 3). The percentage uptake of Tc-99m pertechnetate decreased with age (r = - 0.265; P = 0.034). Although this relationship between the percentage uptake and age was not statistically significant in women (P= 0.365), there was still a negative correlation between these two variables (r = - 0.163). This negative correlation could be attributed to the physiological decrease in iodine uptake, a diminished in thyroid follicle volumes, and the reduction of colloidal content in the elderly (12). This finding further indicates an age-related decrease in Tc-99m pertechnetate uptake, which could be clinically vital in the interpretation of thyroid scintigraphy at different ages. With regard to the small sample size in this study, no definite opinion can be expressed in this respect. 

One of the most conspicuous limitations of the study is its small sample size. Also, the study was conducted in one geographical region in Iran (Mazandaran province). In addition, as the amount of uptake of radiopharmaceuticals such as radioactive iodine and Tc-99m pertechnetate varies in different people based on the amount of iodine intake through food, drugs and supplements, perhaps the uptake pattern of these radiopharmaceuticals varies among population groups in different provinces of Iran. Accordingly, the estimated range of Tc-99m pertechnetate uptake in this study cannot be generalized as a criterion for the whole country. Thus, in the light of this issue, a comprehensive and multi-center study needs to be conducted. The results of the current study may provide valuable information about thyroid treatment and could also exert it influence on the proper handling of thyroid patients. The periodic revalidation of normal reference values is highly recommended. The findings of this study, like those of similar studies, demonstrate a general downward trend in normal thyroid Tc-99m pertechnetate uptake.
